# Host resilience to emerging coronaviruses

**DOI:** 10.2217/fvl-2016-0060

**Published:** 2016-07-01

**Authors:** Amanda M Jamieson

**Affiliations:** 1 Department of Microbiology and Immunology, Brown University, Providence, RI, USA

**Keywords:** ALI, ARDS, coronavirus, disease tolerance, host resilience, MERS-CoV, pulmonary, respiratory infection, SARS-CoV, viral pathogenesis

## Abstract

Recently, two coronaviruses, severe acute respiratory syndrome coronavirus and Middle East respiratory syndrome coronavirus, have emerged to cause unusually severe respiratory disease in humans. Currently, there is a lack of effective antiviral treatment options or vaccine available. Given the severity of these outbreaks, and the possibility of additional zoonotic coronaviruses emerging in the near future, the exploration of different treatment strategies is necessary. Disease resilience is the ability of a given host to tolerate an infection, and to return to a state of health. This review focuses on exploring various host resilience mechanisms that could be exploited for treatment of severe acute respiratory syndrome coronavirus, Middle East respiratory syndrome coronavirus and other respiratory viruses that cause acute lung injury and acute respiratory distress syndrome.

The 21st century was heralded with the emergence of two novel coronaviruses (CoV) that have unusually high pathogenicity and mortality [1–5]. Severe acute respiratory syndrome coronavirus (SARS-Cov) was first identified in 2003 [6–9]. While there was initially great concern about SARS-CoV, once no new cases emerged, funding and research decreased. However, a decade later Middle East respiratory syndrome coronavirus (MERS-CoV), also known as HCoV-EMC, emerged initially in Saudi Arabia [3,10]. SARS-CoV infected about 8000 people, and resulted in the deaths of approximately 10% of those infected [11]. While MERS-CoV is not as widespread as SARS-CoV, it appears to have an even higher mortality rate, with 35–50% of diagnosed infections resulting in death [3,12–13]. These deadly betacoronavirus viruses existed in animal reservoirs [4–5,9,14–15]. Recently, other CoVs have been detected in animal populations raising the possibility that we will see a repeat of these types of outbreaks in the near future [11,16–20]. Both these zoonotic viruses cause a much more severe disease than what is typically seen for CoVs, making them a global health concern. Both SARS-CoV and MERS-CoV result in severe lung pathology. Many infected patients have acute lung injury (ALI), a condition that is diagnosed based on the presence of pulmonary edema and respiratory failure without a cardiac cause. In some patients there is a progression to the more severe form of ALI, acute respiratory distress syndrome (ARDS) [21–23].

In order to survive a given infection, a successful host must not only be able to clear the pathogen, but tolerate damage caused by the pathogen itself and also by the host's immune response [24–26]. We refer to resilience as the ability of a host to tolerate the effects of pathogens and the immune response to pathogens. A resilient host is able to return to a state of health after responding to an infection [24,27–28]. Most currently available treatment options for infectious diseases are antimicrobials, and thus target the pathogen itself. Given the damage that pathogens can cause this focus on rapid pathogen clearance is understandable. However, an equally important medical intervention is to increase the ability of the host to tolerate the direct and indirect effects of the pathogen, and this is an area that is just beginning to be explored [29]. Damage to the lung epithelium by respiratory pathogens is a common cause of decreased resilience [30–32]. This review explores some of the probable host resilience pathways to viral infections, with a particular focus on the emerging coronaviruses. We will also examine factors that make some patients disease tolerant and other patients less tolerant to the viral infection. These factors can serve as a guide to new potential therapies for improved patient care.

## Pathogenesis of SARS-CoV & MER-CoV

Both SARS-CoV and MERS-CoV are typified by a rapid progression to ARDS, however, there are some distinct differences in the infectivity and pathogenicity. The two viruses have different receptors leading to different cellular tropism, and SARS-CoV is more ubiquitous in the cell type and species it can infect. SARS-CoV uses the ACE2 receptor to gain entry to cells, while MERS-CoV uses the ectopeptidase DPP4 [33–36]. Unlike SARS-CoV infection, which causes primarily a severe respiratory syndrome, MERS-CoV infection can also lead to kidney failure [37,38]. SARS-CoV also spreads more rapidly between hosts, while MERS-CoV has been more easily contained, but it is unclear if this is due to the affected patient populations and regions [3–4,39]. Since MERS-CoV is a very recently discovered virus, [40,41] more research has been done on SARS-CoV. However, given the similarities it is hoped that some of these findings can also be applied to MERS-CoV, and other potential emerging zoonotic coronaviruses.

Both viral infections elicit a very strong inflammatory response, and are also able to circumvent the immune response. There appears to be several ways that these viruses evade and otherwise redirect the immune response [1,42–45]. The pathways that lead to the induction of the antiviral type I interferon (IFN) response are common targets of many viruses, and coronaviruses are no exception. SARS-CoV and MERS-CoV are contained in double membrane vesicles (DMVs), that prevents sensing of its genome [1,46]. As with most coronaviruses several viral proteins suppress the type I IFN response, and other aspects of innate antiviral immunity [47]. These alterations of the type I IFN response appear to play a role in immunopathology in more than one way. In patients with high initial viral titers there is a poor prognosis [39,48]. This indicates that reduction of the antiviral response may lead to direct viral-induced pathology. There is also evidence that the delayed type I IFN response can lead to misregulation of the immune response that can cause immunopathology. In a mouse model of SARS-CoV infection, the type I IFN response is delayed [49]. The delay of this potent antiviral response leads to decreased viral clearance, at the same time there is an increase in inflammatory cells of the immune system that cause excessive immunopathology [49]. In this case, the delayed antiviral response not only causes immunopathology, it also fails to properly control the viral replication. While more research is needed, it appears that MERS has a similar effect on the innate immune response [5,50].

## Antiviral treatments & vaccination

The current treatment and prevention options for SARS-CoV and MERS-CoV are limited. So far there are no licensed vaccines for SAR-CoV or MERS-CoV, although several strategies have been tried in animal models [51,52]. There are also no antiviral strategies that are clearly effective in controlled trials. During outbreaks several antiviral strategies were empirically tried, but these uncontrolled studies gave mixed results [5,39]. The main antivirals used were ribavirin, lopinavir and ritonavir [38,53]. These were often used in combination with IFN therapy [54]. However, retrospective analysis of these data has not led to clear conclusions of the efficacy of these treatment options. Research in this area is still ongoing and it is hoped that we will soon have effective strategies to treat novel CoV [3,36,38,40,55–64].

## Host resilience to SARS-CoV & MERS-CoV

The lack of effective antivirals makes it necessary to examine other potential treatments for SARS-CoV and MERS-CoV. Even if there were effective strategies to decrease viral burden, for these viruses, the potential for new emerging zoonotic CoVs presents additional complications. Vaccines cannot be produced in time to stop the spread of an emerging virus. In addition, as was demonstrated during SARS-CoV and MERS-CoV outbreaks, there is always a challenge during a crisis situation to know which antiviral will work on a given virus. One method of addressing this is to develop broad-spectrum antivirals that target conserved features of a given class of virus [65]. However, given the fast mutation rates of viruses there are several challenges to this strategy. Another method is to increase the ability of a given patient to tolerate the disease, i.e., target host resilience mechanisms. So far this has largely been in the form of supportive care, which relies on mechanical ventilation and oxygenation [29,39,66].

Since SARS-CoV and MERS-CoV were discovered relatively recently there is a lack of both patient and experimental data. However, many other viruses cause ALI and ARDS, including influenza A virus (IAV). By looking at data from other high pathology viruses we can extrapolate various pathways that could be targeted during infection with these emerging CoVs. This can add to our understanding of disease resilience mechanisms that we have learned from direct studies of SARS-CoV and MERS-CoV. Increased understanding of host resilience mechanisms can lead to future host-based therapies that could increase patient survival [29].

One common theme that emerges in many respiratory viruses including SARS-CoV and MERS-CoV is that much of the pathology is due to an excessive inflammatory response. A study from Josset *et al.* examines the cell host response to both MERS-CoV and SARS-CoV, and discovered that MERS-CoV dysregulates the host transcriptome to a much greater extent than SARS-CoV [67]. It demonstrates that glucocorticoids may be a potential way of altering the changes in the host transcriptome at late time points after infection. If host gene responses are maintained this may increase disease resilience. Given the severe disease that manifested during the SARS-CoV outbreak, many different treatment options were empirically tried on human patients. One immunomodulatory treatment that was tried during the SARS-CoV outbreak was systemic corticosteroids. This was tried with and without the use of type I IFNs and other therapies that could directly target the virus [68]. Retrospective analysis revealed that, when given at the correct time and to the appropriate patients, corticosteroid use could decrease mortality and also length of hospital stays [68]. In addition, there is some evidence that simultaneous treatment with IFNs could increase the potential benefits [69]. Although these treatments are not without complications, and there has been a lack of a randomized controlled trial [5,39].

Corticosteroids are broadly immunosuppressive and have many physiological effects [5,39]. Several recent studies have suggested that other compounds could be useful in increasing host resilience to viral lung infections. A recent paper demonstrates that topoisomerase I can protect against inflammation-induced death from a variety of viral infections including IAV [70]. Blockade of C5a complement signaling has also been suggested as a possible option in decreasing inflammation during IAV infection [71]. Other immunomodulators include celecoxib, mesalazine and eritoran [72,73]. Another class of drugs that have been suggested are statins. They act to stabilize the activation of aspects of the innate immune response and prevent excessive inflammation [74]. However, decreasing immunopathology by immunomodulation is problematic because it can lead to increased pathogen burden, and thus increase virus-induced pathology [75,76]. Another potential treatment option is increasing tissue repair pathways to increase host resilience to disease. This has been shown by bioinformatics [77], as well as in several animal models [30–31,78–79]. These therapies have been shown in cell culture model systems or animal models to be effective, but have not been demonstrated in human patients. The correct timing of the treatments is essential. Early intervention has been shown to be the most effective in some cases, but other therapies work better when given slightly later during the course of the infection. As the onset of symptoms varies slightly from patient to patient the need for precise timing will be a challenge.

Examination of potential treatment options for SARS-CoV and MERS-CoV should include consideration of host resilience [29]. In addition to the viral effects, and the pathology caused by the immune response, there are various comorbidities associated with SARS-CoV and MERS-CoV that lead to adverse outcomes. Interestingly, these additional risk factors that lead to a more severe disease are different between the two viruses. It is unclear if these differences are due to distinct populations affected by the viruses, because of properties of the virus themselves, or both. Understanding these factors could be a key to increasing host resilience to the infections. MERS-CoV patients had increased morbidity and mortality if they were obese, immunocompromised, diabetic or had cardiac disease [4,12]. Risk factors for SARS-CoV patients included an older age and male [39]. Immune factors that increased mortality for SARS-CoV were a higher neutrophil count and low T-cell counts [5,39,77]. One factor that increased disease for patients infected with SARS-CoV and MERS-CoV was infection with other viruses or bacteria [5,39]. This is similar to what is seen with many other respiratory infections. A recent study looking at malaria infections in animal models and human patients demonstrated that resilient hosts can be predicted [28]. Clinical studies have started to correlate specific biomarkers with disease outcomes in ARDS patients [80]. By understanding risk factors for disease severity we can perhaps predict if a host may be nonresilient and tailor the treatment options appropriately.

## Conclusion & future perspective

A clear advantage of targeting host resilience pathways is that these therapies can be used to treat a variety of different infections. In addition, there is no need to develop a vaccine or understand the antiviral susceptibility of a new virus. Toward this end, understanding why some patients or patient populations have increased susceptibility is of paramount importance. In addition, a need for good model systems to study responses to these new emerging coronaviruses is essential. Research into both these subjects will lead us toward improved treatment of emerging viruses that cause ALI, such as SARS-CoV and MERS-CoV.

**EXECUTIVE SUMMARY**

- Severe acute respiratory syndrome coronavirus and Middle East respiratory syndrome coronavirus are zoonotic coronaviruses that cause acute lung injury and acute respiratory distress syndrome.
- Antivirals have limited effects on the course of the infection with these coronaviruses.
- There is currently no vaccine for either severe acute respiratory syndrome coronavirus or Middle East respiratory syndrome coronavirus.
- Host resilience is the ability of a host to tolerate the effects of an infection and return to a state of health.
- Several pathways, including control of inflammation, metabolism and tissue repair may be targeted to increase host resilience.
- The future challenge is to target host resilience pathways in such a way that there are limited effects on pathogen clearance pathways. Future studies should determine the safety of these types of treatments for human patients.
